# Moonlighting Proteins Shine New Light on Molecular Signaling Niches

**DOI:** 10.3390/ijms22031367

**Published:** 2021-01-29

**Authors:** Ilona Turek, Helen Irving

**Affiliations:** Department of Pharmacy and Biomedical Sciences, La Trobe Institute for Molecular Science, La Trobe University, Bendigo, VIC 3550, Australia; i.turek@latrobe.edu.au

**Keywords:** guanylate cyclase, receptor like kinase, brassinosteroid insensitive 1 (BRI1), phytosulfokine receptor 1 (PSKR1), danger associated peptide receptor (PEPR1 and PEPR2), wall associated kinase like 10 (WAKL10), nanodomains, moonlighting proteins, cryptic enzyme, 3′,5′-cyclic guanosine monophosphate (cGMP)

## Abstract

Plants as sessile organisms face daily environmental challenges and have developed highly nuanced signaling systems to enable suitable growth, development, defense, or stalling responses. Moonlighting proteins have multiple tasks and contribute to cellular signaling cascades where they produce additional variables adding to the complexity or fuzziness of biological systems. Here we examine roles of moonlighting kinases that also generate 3′,5′-cyclic guanosine monophosphate (cGMP) in plants. These proteins include receptor like kinases and lipid kinases. Their guanylate cyclase activity potentiates the development of localized cGMP-enriched nanodomains or niches surrounding the kinase and its interactome. These nanodomains contribute to allosteric regulation of kinase and other molecules in the immediate complex directly or indirectly modulating signal cascades. Effects include downregulation of kinase activity, modulation of other members of the protein complexes such as cyclic nucleotide gated channels and potential triggering of cGMP-dependent degradation cascades terminating signaling. The additional layers of information provided by the moonlighting kinases are discussed in terms of how they may be used to provide a layer of fuzziness to effectively modulate cellular signaling cascades.

## 1. Introduction

Biological systems are best represented by fuzzy logic rather than Boolean logic [1] and this stems from the molecular constituents, to cells, to entire organisms and ecosystems. Unlike Boolean logic, fuzzy logic has variables that can be any real number between 0 and 1 and so reflects the complexities of natural systems. The molecular constituents, particularly proteins in and outside cells, can have variable conformations, states of folding and expression intensities that all contribute to levels of complexity and thus functional “fuzziness” [1,2,3]. Together proteins and complementary molecules enact signaling and metabolic cascades enabling cellular function and communication with other cells in multicellular organisms. Some effects require specific levels of proteins within tightly regulated cellular domains to generate functional outputs. These metabolic and signaling cascades involve networks of proteins interacting closely due to intracellular crowding of proteins and phase separation [4,5,6]. Small molecular products from protein catalytic and signal outputs can act further afield [7,8,9,10]. However, if the small molecules bind to these or nearby proteins, their diffusion is likely to be restricted [5,11,12]. On top of this a further level of complexity exists as proteins can have moonlighting functions [3].

Moonlighting proteins are classified as single chain polypeptides that perform two or more physiologically relevant and distinct functions which do not result from gene fusions, splice variants, or multiple isoforms [3,13,14,15,16]. Combined with an inherent human desire to reduce function to its minimal components, this variety in potential additional functions has obscured the fact that moonlighting activities add an extra layer of complexity to how proteins modulate cellular function. Although these is no straightforward explanation why some proteins acquire multitasking abilities, it can be speculated that conservation of energy contributes to this evolutionary strategy as instead of producing two proteins the task can be completed by one. However, it is important to note that a fraction of moonlighting proteins is evolutionary derived from ancient enzymes—highly conserved proteins present in many different organisms. This not only increases the chance of developing additional functions by these proteins (e.g., through mutations occurring in the course of evolution), but also signifies the importance of the protein multitasking phenomenon.

Moonlighting proteins can be cytosolic enzymes that have another catalytic activity, join another protein or multiprotein complexes, bind additional small molecule ligands, bind nucleic acid chains, or migrate to different intracellular or, in some cases, extracellular locations [3,15,16]. Since the discovery of the first moonlighting protein, duck lens delta-cristallin having argininosuccinate arginine-lyase activity [17], several examples of moonlighting proteins have been articulated, including enzymes acting as transcription factors, chaperones, extracellular growth factors, and cell surface adhesins. Most moonlighting functions of proteins have been found by serendipity and currently there is no straightforward method to delineate moonlighting action of a protein. However, over the last decade significant effort has been made to determine if the protein has the capacity to moonlight. These include analyses of protein sequence and structure and functional sites, conserved motifs and domains, protein-protein interaction patterns, assisted with biochemical methods, to decipher protein structure—function relationships, and extensive attempts to create software tools for prediction and annotation of moonlighting proteins and a database of moonlighting proteins have been made [18,19,20,21,22]. Examples of additional catalytic functions are in general less common in the literature [3,15]. One example is a class of novel guanylate cyclases where a moonlighting or cryptic enzymatic function is buried within a larger catalytic kinase domain [23,24]. This review will focus on normal and moonlighting roles of these kinases and how they may be used to provide a layer of “fuzziness” to effectively modulate cellular signaling cascades.

## 2. Moonlighting Kinases

**Figure 1:**
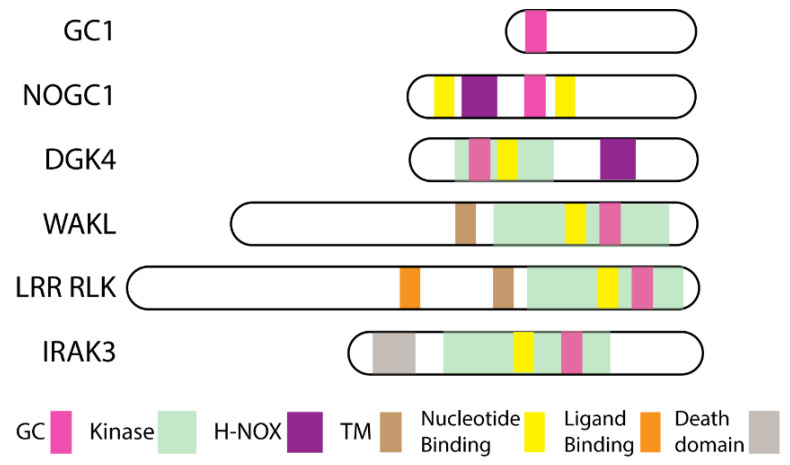
Schematic showing domain architecture of the guanylate cyclases discussed in the text. The guanylate cyclase is depicted in pink, predicted nucleotide binding sites are shown in yellow, kinase domain in pale green, heme-NO/oxygen (H-NOX) domains in purple, transmembrane (TM) domain in brown, ligand binding in orange and the death domain in grey. The schemes are relative to the predicted protein size using the plant leucine rich repeat receptor like kinase (LRR RLK) phytosulfokine receptor (PSKR1) receptor as the reference. The proteins are all plant proteins (GC1 refers to guanylate cyclase 1, NOGC1 as nitric oxide GC1, DGK4 is diacylglycerol kinase 4, and WAKL is wall associated kinase like) except interleukin receptor 1 associated kinase 3 (IRAK3) which is a mammalian protein. IRAK3 contains a kinase homology domain (pseudokinase) differing from LRR RLK, WAKL, and DGK4 which are active kinases.

Moonlighting kinases were first discovered via pattern motif searches for guanylate cyclases in plants. 3′,5′-cyclic guanosine monophosphate (cGMP), the product of guanylate cyclase, occurs in plants although generally at lower levels than in animal systems which generated some controversy about its presence that was resolved through cGMP detection by mass spectrometry [25,26,27,28]. Moreover, cGMP was implicated in mediating hormonal and environmental modulation of physiological responses in plant growth and development [29,30,31,32,33,34]. Therefore, it was surprising that no guanylate cyclases were assigned following the release in 2000 of the full genome sequence of *Arabidopsis thaliana* [35,36]. Furthermore, credible candidates were not identified in Basic Local Alignment Search Tool (BLAST) searches employing ancillary pattern-hit initiated- (phi-), position-iterated- (psi-), and domain enhanced lookup time accelerated (delta-) BLAST with annotated guanylate cyclases from prokaryotes and eukaryotes [35,37,38]. It was hypothesized that amino acids critical to the catalytic action of guanylate cyclases would be conserved in plants like other organisms [36,39]. Hence the pattern search motif was derived from an analysis of lower and higher eukaryote guanylate cyclase enzymes [35] focusing on amino acids conserved in the catalytic function [40,41,42]. The initial search motif revealed seven *A. thaliana* candidates, including two kinases and the first characterized plant guanylate cyclase AtGC1 (AT5G05930) [35]. Since then, orthologs of AtGC1 have been characterized in *Zea mays, Pharbitis nil,* and *Hippeastrum hybridum* [43,44,45] where they have quite diverse roles in modulating pathogen or light responses. Positioning of the guanylate cyclase center varies between known plant guanylate cyclases (Figure 1).

### 2.1. Wall Associated Kinase Like (WAKL)

One of the kinase candidate guanylate cyclases is wall associated kinase like 10 (AtWAKL10, AT1G79680), a member of the wall associated kinase (WAK) clade of the receptor like kinase (RLK) superfamily [46]. WAKs and WAKLs are very strongly connected with cell walls and have an extracellular domain containing epidermal growth factor (EGF) repeat regions, a transmembrane spanning domain and an intracellular serine/threonine kinase domain [47,48,49,50]. WAKs and WAKLs are involved in regulating cell expansion in addition to sensing and responding to abiotic and biotic agents possibly due to sensing pectin fragments [48,51]. The recombinant kinase domain of AtWAKL10 where the guanylate cyclase motif is found has both kinase and guanylate cyclase activity [52] and thus it is a moonlighting kinase. Congruently as cGMP is involved in plant responses to pathogens [53,54,55], transcript expression and functional genomic studies positively implicate WAKL10 in basal and R-gene mediated resistance [52,56]. Intriguingly, the guanylate cyclase activity of Rlm9 protein, a *Brassica napus* homolog of AtWAKL10, appears to be a key component of the hypersensitive response to infection caused by fungal pathogen *Leptosphaeria maculans* carrying the corresponding avirulence gene *AvrLm5-9* [57]. In accordance with this notion, the wheat Stb6 protein lacks the guanylate cyclase in its kinase domain and fails to trigger a hypersensitive response [58]. This may imply the importance of cGMP generated by the moonlighting kinases in biotic responses. Recently a rice (*Oryza sativa*) gene OsWAKL21.2 shown to have dual guanylate cyclase and kinase activity was found to be involved in immune responses in rice and also, when heterologously expressed, in Arabidopsis [59].

### 2.2. Leucine Rich Repeat Receptor Like Kinases (LRR RLK)

Mutation of certain amino acids in the search motif covering the catalytic center of recombinant AtGC1 had no effect on its guanylate cyclase activity [37], suggesting that a relaxed search motif may detect more candidate guanylate cyclases. This was indeed the case as more hits were revealed using the relaxed search motif and these hits included many leucine-rich repeat (LRR) RLKs where the candidate guanylate cyclase center is embedded in the kinase domain as described for WAKL10 [37,38,60,61].

LRR RLKs form the major clade of membrane receptor proteins in plants and most contain an extracellular ligand binding domain, a membrane spanning domain and an intracellular kinase domain [46,62,63,64,65,66,67]. RLK is a large family with over 600 genes in Arabidopsis forming over 2% of the total protein [65]. Plant RLKs form part of the monophyletic RLK and *Pelle* gene family sharing common ancestors with animal receptor kinases; in particular the kinase domain has similarities with *Drosophila melanogaster* Pelle kinase and interleukin 1 receptor associated kinases (IRAK) [46,65,68]. Diversity between the RLK and Pelle clades is massive amongst plants with rice for instance having more than twice the number of RLKs found in Arabidopsis [46,64,65,66,68], but quite limited in animals with four IRAK members in vertebrates [69]. This phenomenon is no doubt due to the need of plants to recognize and respond actively to environmental challenges. Animals can escape these challenges but plants due to their sessile nature need to withstand or tolerate them [46,64,65,66,68,70]. Plant RLK diversification has resulted in great variation in the extracellular domain that contributes to their diverse roles mediating all aspects of plant growth and development and responses to the environment, notably to biotic stresses (for reviews, see for instance: [65,71,72,73]). Many plant RLKs bind endogenous peptide ligands such as phytosulfokine [74,75] or pathogen associated molecular patterns (PAMPs) and damage associated molecular patterns (DAMPs) [73,76]. The degree of peptide signaling in plants is perhaps surprising but can be considered an adaptive evolutionary investment [77]. Recent work has attempted to resolve how extracellular domains of RLKs respond by binding or recognizing PAMPs, DAMPs and other signals contribute to the diversity of plant RLKs at genetic and structural levels [64,78,79]. Phylogenetic analyses reveal that the kinase domain is relatively conserved compared to the extracellular domain of RLKs including LRR RLKs [62,63,64,66]. Therefore, it is of interest that some members of the LRR RLK family contain candidate guanylate cyclase centers embedded in the kinase domain, but most do not.

Several of the LRR RLK candidate guanylate cyclases (moonlighting kinases) identified in Arabidopsis [37] have since been shown to contain both kinase and guanylate cyclase activity at least in vitro. These include the brassinosteroid receptor, brassinosteroid insensitive 1 (AtBRI1; AT4G39400) [37,80], phytosulfokine receptor 1 (AtPSKR1; AT2G02220) [81,82,83], the DAMP receptor for Arabidopsis plant elicitor peptide 1 (AtPEP1), AtPEPR1 (AT1G73080), and its tomato homologs SlGC17 and SlGC18 [84,85]. BRI1 and PSKR1 are both important in plant growth, while PEPR1 is involved in plant immunity. Below we briefly describe the function of these molecules with guanylate cyclase activity.

BRI1 was first identified as being critically important in elongation growth [86] and is part of clade Xb of the LRR RLKs [87]. Since, a recent extensive review explores how BRI1 signaling interconnects with plant growth and environmental challenges [88], we only briefly review BRI below. BRI1 recognizes brassinosteroids and forms heterodimers with BRI1-associated kinase (BAK1)/somatic embryogenesis receptor like kinase 3 (SERK3) [79,89,90,91]. Application of exogenous brassinosteroid elevates both colocalization of BRI1 and BAK1/SERK3 and receptor hetero-oligomerization in the plasma membrane of Arabidopsis root epidermal cells, while populations of BRI1 and BAK1/SERK3 colocalized independently of the ligand [92]. Heterodimerization is followed by extensive auto- and trans-phosphorylation [93] and phosphorylation of BRI1 substrate kinases (BSK) [94,95] eventuating in induction of transcription factors inducing expression of genes involved in cell elongation [89]. Interestingly, only about 10% of receptors present at the plasma membrane need to be occupied to stimulate root growth [96]. In addition, stimulation with brassinosteroids can induce increases in cGMP in leaf mesophyll protoplasts or root tips [24,97] but this was not seen with root protoplasts [98]. This disparity in outcomes may be due to differences in assessing cGMP amounts. The cellular increases in cGMP were measured with antibody-based cGMP detection methods and in transgenic FlincG Arabidopsis seedlings [24,97] while root protoplasts were transiently transfected with FlincG [98]. FlincG is a chimeric fluorescent reporter containing the regulatory domain of type 1 protein kinase G (cGMP-dependent protein kinase; PKG) fused in tandem to the circular permuted enhanced green fluorescent protein that was developed initially for animal systems [99] and adapted to plant systems [100]. However, BRI1 downstream signaling appears to involve cGMP. Brassinosteroid-induced transcripts are reduced in the presence of guanylate cyclase inhibitors while mutation of *cyclic nucleotide gated channel 2 (CNGC2)* gene abolished the cGMP-dependent cytosolic release of Ca^2+^ upon brassinosteroid perception [97]. BSK1, a substrate of BRI1, is part of the rapidly activated cGMP phosphoproteome [80,101]. Recombinant kinase domains of BRI1 can generate cGMP [37,80], although this finding has been questioned as other studies using less sensitive methods have not detected cGMP [102]. The BRI1 kinase activation loop is critical for peptide substrate binding and displays structural features reminiscent of both serine/threonine and tyrosine kinases, yet it is still unclear whether the receptor can switch between these activities [102] and if cGMP is involved in this process. Interestingly, mutations in the kinase domain modulate the guanylate cyclase activity [80] suggesting that intramolecular cross-modulation occurs. BRI1 kinase activity enhances guanylate cyclase activity while kinase activity itself is suppressed by cGMP [80] suggestive of a very localized effect by this moonlighting enzyme. An increasing body of evidence suggests BRI1 has functions that are independent of classical brassinosteroid signaling outputs mediated by the canonical brassionsteroid signaling pathway, that are mediated by receptor-like protein 44 (RLP44), which probably acts as a scaffold promoting association of BRI1 and BAK1/SERK3 [103]. RLP44 is under transcriptional control of BRI1 and is able to promote activity of a complex containing PSKR1, through an analogous scaffolding mechanism as observed for the activation of brassinosteroid signaling [104], with BRI1 and PSKR1 likely competing for RLP44 [105]. This is of interest as both BRI1 and PSKR1 contain moonlighting guanylate cyclase function that may result in cGMP-enriched scaffold complexes.

PSKR1 is one of the two Arabidopsis receptors for the sulfated pentapeptide phytosulfokine [106,107] and was identified through elegant ligand binding studies [74]. PSKR1 is a member of the clade Xb of LRR RLKs [87] and has diverse roles in modulating cell expansion, cell differentiation, and plant immunity [23,108]. Phytosulfokine binding to the extracellular island domain in PSKR1 allosterically alters the entire receptor conformation enhancing heterodimerization with SERKs including BAK1/SERK3 [109]. A membrane associated complex forms involving H^+^ ATPases AHA1 and 2, and CNGC17 [110]. Auto-phosphorylation of PSKR1 occurs and additional molecules are phosphorylated [82,111,112] but the downstream signal pathway is unclear. Overexpression of PSKR1 in protoplasts leads to 20-fold increases in cGMP levels in protoplasts, and wildtype protoplasts show a transient increase in cGMP levels in response to the active phytosulfokine ligand [81]. Involvement of phytosulfokine-PSKR1 signaling in plant immune responses was suggested by observations that genes encoding phytosulfokine precursors [113], processing enzymes [114] and PSKR1 receptor [107,115,116] are induced by wounding, several bacterial and fungal elicitors and necrotrophic pathogen *Botrytis cinerea*. Arabidopsis *pskr1* mutant plants displayed growth inhibition, enhanced defense gene expression and enhanced immune responses to elf18 and virulent bacterial pathogen *Pseudomonas syringae* [115]. This is in line with a notion that PSKR-mediated signaling attenuates immune responses and it was proposed that cGMP is the signaling component immediately downstream of PSKR1 [115]. Elevation of cytosolic Ca^2+^ due to phytosulfokine-PSKR1 interaction results in auxin-dependent immunity of tomato plants against *B. cinerea*, and exogenous application of phytosulfokine enhances resistance, while silencing of PSKR1 raises their susceptibility to this fungal pathogen (Zhang et al., 2018). Of note, increases in calcium ions at physiologically likely levels inhibit kinase activity of the recombinant kinase domain while promoting cGMP production [83] and cGMP inhibits kinase activity [81] providing further support that these moonlighting enzymes support a localized effect [23].

AtPEPR1 was identified via a series of cross-linking studies with the endogenous Arabidopsis plant elicitor peptide 1 (AtPEP1), a degradation product of the C terminal of precusor of peptide 1 (PROPEP1) [117], and is a member of clade XI of the LRR RLKs [87]. AtPEPs comprise a family of eight members [118] that mature from their AtPROPEP precursors [119]. Expression of some PROPEPs and both PEPRs is induced by perception of microbe-associated molecular patterns (MAMPs) and other molecules, including methyl jasmonate and ethylene [119,120]. Plant elicitor peptide (PEP) signals are generated rapidly (30 s) following wounding via cleavage of PROPEP through calcium dependent activation of the proteases, metacaspases [121], where they cause medium alkalinization [122] and act as DAMP signals. PEPR-mediated signaling is involved in immunity towards pathogens with lifestyles ranging from hemibiotrophic to necrotrophic [123,124,125,126,127]. AtPEPs amplify defense responses via initiating jasmonic acid [123], ethylene [124,126,128], salicylic acid, Ca^2+^ and hydrogen peroxide [118,120,122,128] signal cascades. AtPEPR1 and AtPEPR2 share structural and functional similarity to the flagellin receptor Flagellin-Sensing2 (FLS2) and the elongation factor Tu (EF-Tu) receptor [117,120,128] and are coupled with BRI1-associated receptor kinase 1 (BAK1) [129,130] and BAK1-Like1 [131], and disruption of BAK1 sensitizes PEPR signaling [127]. The kinase domain of PEPR1 interacts with and directly phosphorylates the receptor-like cytoplasmic kinase Botrytis-induced kinase 1 (BIK1) required for PEP1-induced resistance against *B. cinerea*, while ET-induced expression of defense genes and resistance to *B. cinerea* are compromised in the *pepr1/pepr2* plants [124]. The downstream events are complex as BIK1 also undergoes monoubiquitination to mediate immune signaling [132]. Exogenous PEPs activate mitogen-activated protein kinase 3 (MPK3) and MPK6 [118].

The PEP1-PEPRs system intersects both with auxin and ROS signaling, inhibiting root growth [133,134]. It is noteworthy that PEP1 and both jasmonic acid and auxin have been shown to stimulate increases in cGMP levels using the FlincG reporter system [98,125]. Although the PEP–PEPR system is principally considered as part of the plant defense response, it is also involved in plant development and reproduction [118] and stress tolerance [135]. Both PEPR1/2 carry a guanylate cyclase catalytic center with conserved residues crucial for catalysis embedded in its kinase domain [84,120], but so far only the enzymatic activity of PEPR1 has been experimentally demonstrated [84]. Recombinant protein studies have demonstrated that AtPEPR1, *H. hybridum* HhPEPR1, and tomato homologs SlGC17 and SlGC18, can all generate cGMP [84,85,136]. Downstream signaling following PEP activation involves characteristic pattern induced responses including increased gene transcription that is enhanced in the presence of calcium possibly entering cells by cyclic nucleotide gated channels (CNGC) [84,125]. It is tempting to speculate that PEPR1 forms a complex with CNGCs where localized cGMP generated by PEPR1 can activate CNGCs. Interestingly, PEPR signaling has recently been reported to function downstream of CNGC19, which is activated by elicitors in the cell wall of *Piriformospora indica* and known to be involved in AtPEP1-induced elevation of cytosolic calcium ions [137], and modulate CNGC19-mediated basal immunity to regulate colonization of the fungus in Arabidopsis roots [138].

Several other LRR RLKs were found to contain a guanylate cyclase center in the relaxed searches [37] including ERECTA (ER), ER-like 1 and 2 (ERL1, ERL2), and CLAVATA 1 (CLV1). Like many other LRR RLKs, these molecules have roles in plant growth and development including plant immunity. To date, their guanylate cyclase activity has not been explored. However, some of these molecules have been shown to have links to cGMP production. Although CLV1 has not yet been shown to directly generate cGMP, its peptide ligand CLV3 induces increases in cGMP when applied to root tips of transgenic FlincG Arabidopsis seedlings [139].

### 2.3. Nitric Oxide (NO)-Responsive Moonlighting Proteins

In total, two sensors of nitric oxide (NO) in plants have been identified through pattern searches that recognized separate amino acid sequences in these proteins for both heme-NO/oxygen (H-NOX) binding and guanylate cyclase centers [140,141] that both contain guanylate cyclase centers (Figure 1). The first identified NO-dependent guanylate cyclase 1 (AtNOGC1; AT1G62580) protein is a flavin dependent monooxygenase, which has a higher affinity for NO over O_2_ where NO induces cGMP production [141]. Similar pattern searches predicted that diacylglycerol kinase 4 (AtDGK4; AT5G57690) found predominantly in pollen tubes also contained H-NOX and guanylate cyclase center motifs [61,140]. The two independent groups showed that AtDGK4 is important for growth and directional responses of pollen tubes and AtDGK4 also generates cGMP which like NO can inhibit the kinase activity [142,143]. Diacylglycerol kinases are cytoplasmic atypical (or lipid) kinases that phosphorylate diacylglycerol forming phosphatidic acid and are important in lipid metabolism necessary for plasma and endomembrane signaling. AtDGK4 is an example of a plant cytoplasmic moonlighting kinase as it has both kinase and guanylate cyclase activity [142,143].

## 3. Moonlighting Kinase Guanylate Cyclase Centers

**Figure 2:**
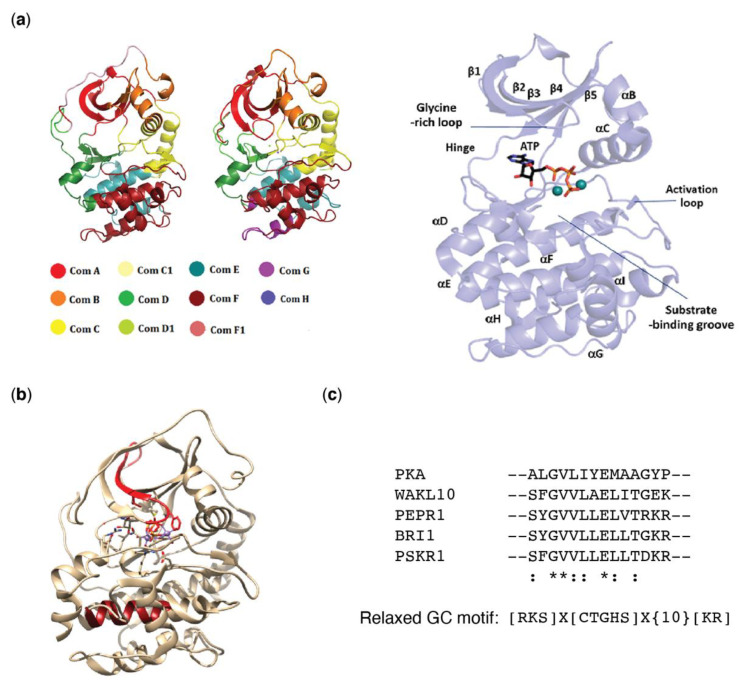
Protein kinase community maps and guanylate cyclase centers. (**a**) The different community maps present in protein kinases depicted using protein kinase A (PKA) (reproduced with permission from [148]). (**b**) Three-dimensional structure of *Mus musculus* PKA (Protein Data Bank entry: 3FJQ, 10.2210/pdb3FJQ/pdb) showing the ATP binding site, position of the guanylate cyclase center of diacylglycerol kinase 4 (DGK4; red) and the guanylate cyclase center of the receptor like kinases (RLK; dark red). (**c**) Alignment of the RLK guanylate cyclase center with the corresponding region in *M. musculus* PKA with amino acids that are identical indicated by asterisk(*), and those that are similar by colon (:).

Kinases play incredibly important parts in cell signaling regulating signal cascades, metabolic pathways, transcription events, and cell cycles. The superfamily of eukaryotic protein kinases contains 12 specific sequence motifs or conserved subdomains (linearly numbered I–XI, inclusive of VIa and VIb) [144,145]. These motifs are scattered throughout the conserved structural core of eukaryotic protein kinases. In their folded state kinases have the superficial structure of a violin where the linear sequence folds over so that it can dynamically and allosterically relay conformational changes to non-linear but adjacent folded parts [146,147,148,149,150]. The fact that changes in other regions of the kinase molecule can allosterically induce dynamic molecular vibrations across the kinase has furthered the allusion to violins where disturbance at one point will induce different tonal and conformational responses at other points [148]. An algorithm developed to identify community boundaries based on central community indices in biological and social networks [151] was used to identify community maps in protein kinases [150]. Community maps (Com A through to Com H) are used to describe each of the three-dimensional folded regions containing 40–60 amino acids of the kinase [146,147,148,150] (Figure 2a). Surprisingly, as their sequence differs, atypical kinases which include the lipid kinases also form similar core folds with an overall similar structure [152].

Of relevance here, the guanylate cyclase center in the RLKs (e.g., BRI1, PSKR1, PEPR1, and WAKL10) is found in domain IX and arises from an α-helix to random loop in the C-lobe [38,60,153] (Figure 2b,c). Changes in this lobe are predicted to modulate the catalytic site via allosterically modulating ComC containing the catalytic site [148,150]. On the other hand, the guanylate cyclase center of the lipid kinase DGK4 is found in domain I of the N-lobe [140,143] (Figure 2b) and is also likely to impinge on substrate binding and phosphorylation actions. These differences in location of the guanylate cyclase center are of interest as they may pertain to their molecular action. However, if dynamic modulation of the localized regions or community maps of the molecule is the key role of the guanylate cyclase component, the regional positioning may not be so important as molecular tuning will effectively occur across the molecule altering kinase activity.

Providing supplementary means of tempering kinase activity will add an extra layer of “fuzziness” to downstream signal interactions smoothing cell function and potentially generating enriched signal niches or nanodomains. Molecular cross talk involving kinase and guanylate cyclase activity is at least important for the function of BRI1 and PSKR1 [80,82]. Kinase dead mutants of BRI1 have reduced guanylate cyclase activity due to either lack of kinase activity or phosphorylated residues [80]. It is conceivable that it is the number of phosphorylated residues as mutations mimicking phosphorylated residues were more effective in modulating guanylate cyclase activity of PSKR1 than those modulating kinase activity [82]. Number of phosphorylated residues has long been recognized as a key tuning event in regulating kinase activity [149]. However, mutations in the guanylate cyclase center do not affect kinase activity of PSKR1 [83]. Small increases in cGMP levels are sufficient to decrease kinase activity in BRI1, PSKR1, and PEPR1 [80,81,154]. DGK4 is an interesting molecule as its kinase activity is reduced by cGMP and also NO—both compounds that DGK4 can itself generate [142,143]. These findings involving intramolecular communication support a role for cGMP in dynamically and allosterically modulating the kinase (discussed further in Section 4).

Phosphorylation action of kinases is their main function, but interest has also developed in their non-catalytic functions as the kinase structure supplies a scaffold that contributes to functions of pseudokinases [155,156,157,158]. At least one eukaryotic pseudokinase, IRAK3 has a functional guanylate cyclase center that contributes to its activity [159]. IRAK3 is involved in regulating the innate immune system in animals [160,161]. The IRAK3 pseudokinase domain is located between an N-terminal death domain and before C-terminal domain (Figure 1). Homology modelling indicates that the pseudokinase domain forms similar fold patterns to models of the kinase domain of PSKR1 with the guanylate cyclase center in a similar position [153]. Phosphorylation status and kinase activity do not contribute to the guanylate cyclase activity although small amounts of cGMP are important in promoting the ability of IRAK3 to suppress nuclear factor kappa B (NFκB) activity [159]. Potentially, localized small amounts of cGMP allosterically modulate IRAK3 conformation to promote down-stream effects.

## 4. Nanodomains Surrounding Moonlighting Kinases

Nanodomains are the immediate areas surrounding the proteins, protein complexes, and other intracellular or extracellular structures such as lipid rafts in membranes. Advances in microscopy techniques have allowed analysis of fluorescent protein (FP) tagged membrane molecules such as BRI1-GFP at the nanoscale using single particle tracking. BRI1 partitions into distinct plasma membrane nanodomains that are important for receptor endocytosis and exocytosis processes [162,163]. The majority of surface expressed BRI1 is not involved in signaling [96] and these receptors have limited plasma membrane mobility [162,163]. Conflicting results have been reported following ligand binding that may be dependent on the cell type and the type of microscopy analysis. In root tips, brassinosteroid activation promoted movement of BRI1 to nanodomains and association with the nanodomain marker flotillin 1 (FLOT1) using variable angle total internal reflection fluorescence (TIRF) microscopy [163]. In cotyledon or leaf epidermal cells, BRI1 was stabilized in specific clusters associated with plant nanodomain marker remorin proteins [162]. Interestingly there are separate clusters of BRI1 and flagellin sensing 2 (FLS2) occurring in the plasma membrane that each contain SERK signaling molecules [162]. In plants protein and lipid movement in the plasma membrane is constrained by interactions with proteins in the cytoskeleton and the cell wall which may act like a cellular exoskeleton [164]. In their recent review [164], Jallais, and Ott discuss implications of lipid and protein interactions with cell wall and cytoskeleton in terms of specific receptor nanodomains highlighting unique features for plants in terms of signaling outcomes.

Moonlighting kinases such as WAKL10, BRI1, PSKR1, and PEPR1 are all examples of RLKs that localize in specific regions in plasma membranes leading to signaling clusters. This is certainly the case for BRI1 [162,163]. So how can this potential amplification due to receptor clustering be further enhanced intracellularly to relay signals. Each of these molecules is a kinase coupled receptor and therefore phosphorylation signaling cascades form an important part of the signal relay system. Phosphorylation has long been recognized as changing the nanodomain near the phosphorylated residue and contributing to protein function by generating an acidic region that modifies protein conformation [165]. To create major conformational changes often more than one residue is phosphorylated and additional kinases may be needed to sequentially phosphorylate the recipient proteins to spark new signal cascades [149,166].

Effects of cGMP are likely to be more subtle but will still induce conformational changes that may alter disordered regions of the protein in question. Such events may be dependent upon the phosphorylation state of the intracellular kinase domain as suggested by findings associated with interactions between kinase and guanylate cyclase activity seen in PSKR1 and BRI1 [80,82,83]. Guanylate cyclase activity of moonlighting kinases has mainly been determined using recombinant kinase domains or even ~100 amino acid fragments containing the guanylate cyclase center. Typically, this guanylate cyclase activity is very low, thereby raising questions about the biological significance of the small amounts of cGMP generated which have been discussed [25,61,80,102,167,168]. Wild type recombinant proteins containing the kinase domain of PSKR1 or BRI1 have greater guanylate cyclase activity than proteins expressing point mutations in the guanylate cyclase center predicted to reduce activity [80,81,83]. Generally mass spectrometry measurements detect greater amounts of cGMP production from the recombinant proteins than antibody-based detection measures [25,80]. Higher guanylate cyclase activities have been observed with recombinant *P. nil* and *H. hybridum* orthologs of GC1 (PnGC1, HhGC1) and HhPEPR1 where variations in the buffer composition have been used including both magnesium and manganese ions [43,44,136] while calcium ions promote guanylate cyclase activity of the recombinant PSKR kinase domain [83].

An additional consideration may be that only small amounts of cGMP are needed to regulate proteins in the immediate clustered receptor nanodomain and so enrich it and stimulate signaling events. The localized nature of cryptic enzyme generated nanodomains due to a lack of realization of receptor clusters has led to this aspect of signal transduction being previously overlooked. Nevertheless, we all recognize that transient and spatially controlled levels of signaling molecules are necessary to generate appropriate responses to environmental and developmental stimuli within defined cytoplasmic areas or cellular compartments. Therefore, cells need localized control mechanisms or “traffic lights” to guide correct outcomes to external stimuli. Such traffic lights include localized spatial changes in Ca^2+^ concentration and localized changes in phosphorylation, as well as changes in cyclic nucleotides or combinations thereof [23,25,164,169,170]. Unregulated universal production of cyclic nucleotides, such as cGMP due to overexpression of mammalian soluble guanylate cyclase resulting in 50–250 fold higher cGMP levels than normal, significantly affect cellular redox state, potentially due to the cross-talk between cGMP and the glutathione redox system [171]. It also results in extensive changes in gene expression and inappropriate protein expression [171] likely leading to many additional protein misfolding events [2].

**Figure 3:**
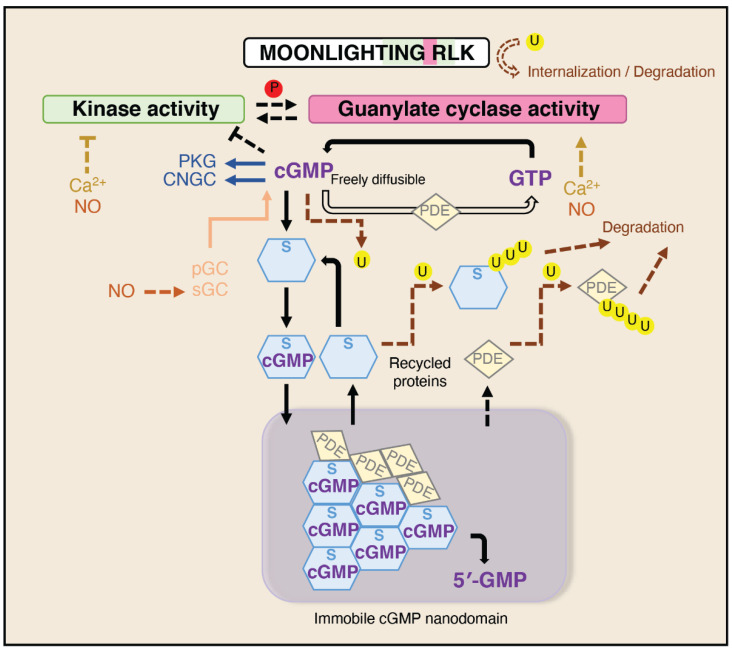
Schematic showing generation of cGMP by a moonlighting receptor like kinase (RLK) and the associated protein complex enabling cGMP enrichment due to a combination of phase condensation of proteins with disordered states and proteins with cGMP binding sites that occurs in the nanodomain surrounding the RLK complex. cGMP—3′,5′-cyclic guanosine monophosphate; GTP—guanosine-5′-triphosphate; 5′-GMP—guanosine 5′-monophosphate; Ca^2+^—calcium; CNGC—cyclic nucleotide gated channel; NO—nitric oxide; PDE—cyclic nucleotide phosphodiesterase; PKG—protein kinase G/cGMP-dependent protein kinase; S—scaffold protein. The letter P in circle indicates phosphate group, while letter U indicates ubiquitin. Arrows indicate positive regulation, while blunt-ends indicate inhibition. Regular lines indicate experimentally confirmed processes, while dashed lines indicate hypothetical actions and processes relating to a subset of the molecules.

We suggest that proteins containing cryptic enzymatic activities, such as the guanylate cyclase in WAKL10, PSKR1, BRI1, and PEPR1 generating cGMP-enriched nanodomains, are part of the solution to highly spatially differentiated stimulus-specific cellular signaling and form a new paradigm in cellular signaling and homeostatic responses [23]. Interestingly, auto-generation of a cGMP-enriched nanodomain is part of the mechanism of action of the cytoplasmic protein IRAK3, which is involved in inhibiting animal cell responses to DAMPs [153,159], indicating that this is potentially a universal paradigm in cell signaling. Establishment of a cGMP-enriched niche or nanodomain in the vicinity of the protein is a puzzle as physics suggests that small molecules will rapidly diffuse away unless they are attached in some way [172]. Once stated, the obvious answer is that cGMP must be attaching to either moonlighting kinases generating cGMP or to other members of the localized signaling interactome. Dynamic cyclic adenosine monophosphate (cAMP) nanodomains have recently been visualized in animal cells where cAMP leaving the nanodomain is rapidly converted to AMP by localized cyclic nucleotide phosphodiesterases (PDE) [173], enzymes that hydrolyze cyclic nucleotide monophosphate (cNMP) to 5′-nucleotide monophosphate. In addition, cAMP-dependent protein kinase A type I regulatory subunit forms biomolecular condensates enriched in cAMP and active kinase [174]. Phase condensation of proteins involves interactions with disordered protein states and is associated with accumulation of specific small molecules such as drugs and cAMP [174,175]. In combination with protein binding sites, phase condensation provides mechanisms of concentrating small molecules (Figure 3). Plant PDEs degrading cGMP are elusive, as earlier bioinformatic searches failed to identify homologs of animal PDEs. However, PDE activity has been detected in crude protein extracts from different plant species in the 1970s [176] and partially purified PDE from chloroplasts of *Phaseolus vulgaris* displayed enzymatic activity in the presence of cAMP and cGMP [177]. A novel cGMP-activated PDE encoded by an ancient gene not represented in animals, is encoded in Arabidopsis by *PDE1* [178], and more plant PDEs remain to be discovered and characterized. Numerous analogies between cGMP– and cAMP–PDE signaling in plants and animals make it likely that PDE-mediated degradation of cGMP and formation of condensates facilitating cGMP enrichment with scaffold proteins and decomposition by PDE occurs in plants in a similar manner as it has been shown in animals (Figure 3).

Initially bioinformatic studies were undertaken to identify plant cyclic nucleotide binding proteins looking for the evolutionary conserved cyclic nucleotide binding domain [179] and the GAF domain (named for cGMP-regulated cyclic nucleotide phosphodiesterases, some adenylate cyclases, and the bacterial transcription factor FhlA) [180]. Plant proteins with predicted cyclic nucleotide binding domains were shaker type potassium channels, CNGCs and acyl-CoA thioesters [181]. Experimental evidence supports cyclic nucleotide regulation of shaker type potassium channels and CNGCs [182,183]. CNGC2 (also known as defense no death1, DND1), conducts monovalent cations but excludes Na^+^ [184] and is activated by cAMP to conduct calcium ions into cells downstream of nitric oxide production stimulating hypersensitivity plant immune responses [185]. CNGC4 (also known as DND2) is K^+^ and Na^+^-permeable channel implicated in a signaling pathway leading to hypersensitive responses, and is more efficiently activated by cGMP than cAMP [186]. CNGC2 and CNGC4 assemble into functional calmodulin-gated calcium channels phosphorylated by BIK1 in response to flg22, bridging the gap between the pattern-recognition receptor complex and Ca^2+^-dependent programs in the PAMP-triggered immunity [187]. Interestingly, PSKR1 (and potentially other RLKs) and BAK1 assemble with CNGC17 and AHA1 and 2 [110]. BAK1 also phosphorylates and regulates stability of CNGC19 and CNGC20 that form a heteromeric Ca^2+^-permeable channel and contribute additively to *bak1*/*serk4* cell death [188]. Rice OsCNGC9 calcium channel is activated through phosphorylation by PAMP-triggered immune-related receptor-like cytoplasmic kinase (RLCK) OsRLCK185 and regulates resistance to rice blast disease [189]. All these observations provide fuel for speculation that RLKs [190] and possibly moonlighting proteins are kinases that phosphorylate CNGCs and it would be interesting to investigate the impact of cGMP concentration in the vicinity of such a nanocluster on this process. cGMP (with a modest effect of cAMP) stimulates nonselective Ca^2+^-permeable cation channel activity of CNGC5 and CNGC6 in guard cells, while mutations in *CNGC1, CNGC2, or CNGC20* failed to disrupt cGMP-activated currents [191].

In addition, phytochrome proteins and ethylene receptors were predicted to contain GAF domains [181]. The GAF domains may be functional in phytochromes where cGMP is involved in their signaling [192,193,194] but structural studies suggest this may be unlikely [195]. More recently, an affinity purification strategy was used to purify soluble cyclic nucleotide binding proteins from Arabidopsis where eight proteins with cyclic nucleotide binding or GAF domains that had not been annotated in the databases and four other cyclic nucleotide binding proteins were identified [196]. Perhaps surprisingly several of the proteins are enzymes in carbon fixation pathways (e.g., phosphoglycerate kinase 1 (PGK1), glyceraldehyde-3-phosphate dehydrogenase B subunit (GAPB), transketolase (TKL), carbonic anhydrase 1 (CA1), serine hydroxymethyltransferase 1 (SHMT1), and glycolate oxidase 1 (GOX1)) that appear to be directly modulated by cyclic nucleotides and also involved in H_2_O_2_ signaling defense responses [196]. These findings highlight the connections between metabolism and moonlighting proteins. For instance, enzymes in the Kreb’s or citric acid cycle exhibit promiscuity that may be involved in regulating potential metabolic damage [197], but enzymes such as aconitase have separate moonlighting roles [198,199]. Cytochrome *c* involved in the vital function of mitochondrial respiration also is a key player in apoptosis and the formation of the apoptosome [200]. These additional functions of proteins involved in metabolism contribute to the overall fuzziness associated with cell regulation [3].

The need for localized nanodomain signal niches involving cGMP is perhaps emphasized by the diverse range of responses dependent on cellular changes in cGMP. Plant growth and development involves alterations in hormones such as auxin, cytokinin, gibberellic acid and plant natriuretic peptide that mediate increases in cellular cGMP [29,32,34,98,201]. At the cellular level these involve changes in ion movement, phosphoproteome and transcriptome [98,101,202]. Localization of these responses to specific regions containing moonlighting kinases (and other guanylate cyclases such as GC1) may be one way to constrain signaling cascades to reflect the primary ligand. Such cGMP enriched nanodomains likely involve protein phase condensation of complexes of proteins with cGMP binding sites (Figure 3).

## 5. Degradation of Moonlighting Proteins

Although protein turnover and degradation are critical to the signaling competence of moonlighting RLKs and plant cellular homeostasis, little is known about the role of ubiquitination on their function, the determinants of recycling or degradation, and a possible contribution of autophagy [203,204]. Nor is it known if the kinase or guanylate cyclase activity of moonlighting proteins have a potential impact on those activities. Clathrin-mediated endocytosis is the major internalization route of many plasma membrane proteins, including PEPR1 and BRI1 [205,206,207]. In contrast to PEPR1 where functional endocytic machinery is important for downstream signaling activation [206,207], endocytosis of BRI1 is mainly required for signaling attenuation [205] and is largely independent of the presence or absence of brassinosteroids [208]. Insight into turnover and degradation of plant receptor kinases is an emerging field, and ubiquitination of several moonlighting proteins, such as BRI1 [209,210], PSKR1, and ERECTA [211] have already been reported. Nevertheless, the process is far from being understood and extensive effort is required to decipher which ubiquitin ligases and ubiquitin conjugating enzymes are needed for internalization and sorting of most of the moonlighting proteins.

In vivo, BRI1 is post-translationally modified by K63 polyubiquitin chains and its ubiquitination promotes BRI1 internalization from plasma membrane and is crucial for its recognition at the trans-Golgi network and early endosomes for vacuolar targeting, while loss of BRI1 ubiquitination at residue K866 is associated with subtle brassinosteroid hypersensitivity [209]. Although BRI1 ubiquitination is largely independent of ligand binding, it requires BRI1 kinase activity and the presence of BAK1/SERK3, which are dependent on brassinosteroids [209]. Ligand bound BRI1 is internalized through an endocytic pathway mediated via plant U-box (PUB) E3 ubiquitin ligases PUB12 and PUB13 leading to its degradation [210] and terminating the signaling process. Phosphorylation of PUB13 mediated by this moonlighting kinase regulates association of BRI1-PUB13 complex [210], suggesting an intertwined regulation of these two proteins. Hence, it would be interesting to investigate whether the guanylate cyclase activity of BRI1 may affect its ability to be ubiquitinated, as cGMP has been reported to decrease BRI1 kinase activity [80]. If so, then the next question is whether the plasma membrane pool of BRI1, its degradation and its interaction with its ubiquitin ligases, is modulated by cGMP.

Apart from enhancing proteasomal degradation (but not autophagy) by activating cGMP-dependent protein kinase G (PKG), cGMP has been shown to rapidly stimulate ubiquitin conjugation, thus increasing cellular levels of polyubiquitinated proteins, and degradation of both short-lived and long-lived cell proteins in animals [212]. These rapid (within 5 min) increases are unlikely to result from gene expression and point at site-specific action of cGMP [212]. Thus, speculations on the potential function of guanylate cyclase activity of plant moonlighting kinases in stimulating ubiquitination, quality control and degradation of distinct populations of proteins in the vicinity of moonlighting kinases can be contemplated. In analogy to mammalian cells, 8-bromo-cGMP enhances auxin-induced degradation of Aux/indole-3-acetic acid (IAA) protein modulated by the transport inhibitor response 1 (TIR1) ubiquitin-proteasome pathway [213]. Although 8-bromo-cGMP is unable to directly influence the auxin-dependent TIR1-Aux/IAA interactions [213], the cGMP-mediated modulation of auxin signaling through cGMP-dependent protein kinase was proposed. Therefore, the product of the guanylate cyclase activity of plant moonlighting kinases potentially can accelerate protein degradation rates in a proteasome-independent manner. cGMP also enhances ATP-dependent proteasome activity, while the inhibition of cGMP synthesis inhibits degradation of Aux/IAA protein [213]. Thus, cGMP may operate in multiple ways, including the proteasome-dependent and independent mechanisms of modulating protein degradation.

## 6. Conclusions

Plant moonlighting kinases, described here, have significant roles in their kinase form that contribute to whole plant regulation. These proteins are involved in multiple signal pathways and the network of signaling interactions can potentially be subtly modulated via the guanylate cyclase activity that they exhibit. The end-product cGMP not only directly modulates kinase activity but also is involved in modulating interacting proteins such as CNGCs and potentially stimulating protein ubiquitination. We argue that moonlighting kinases create a cGMP-enriched niche that will modulate the immediate interactome providing an overlay to the signaling events important in developing localized intracellular regions of fuzziness, ensuring that asymmetrical response can occur that are essential in plant growth and development. Investigating this hypothesis involves visualizing and monitoring development of signal niches or nanodomains and correlating these with subtle alterations in plant growth and development.

## Data Availability

No new data were created or analyzed in this study. Data sharing is not applicable to this article.
